# Center Degenerated Walking-Primer PCR: A Novel and Universal Genome-Walking Method

**DOI:** 10.3390/cimb47080602

**Published:** 2025-08-01

**Authors:** Dandan Gao, Zhenkang Pan, Hao Pan, Yinwei Gu, Haixing Li

**Affiliations:** 1 Key Laboratory of Biotechnology and Bioengineering of State Ethnic Affairs Commission, Biomedical Research Center, Northwest Minzu University, Lanzhou 730030, China; gaodan0322@163.com; 2 State Key Laboratory of Food Science and Resources, Nanchang University, Nanchang 330047, China; pzk2894866116@163.com (Z.P.); 18879271997@163.com (H.P.); guyinwei2022@163.com (Y.G.); 3 Sino-German Joint Research Institute, Nanchang University, Nanchang 330047, China

**Keywords:** genome-walking PCR, normal walking primer, center degenerated walking primer, partial annealing, gene-specific primer

## Abstract

Enhancing the specificity and applicability of PCR-based genome-walking methods is highly desirable. A new and universal genome-walking tool, called center degenerated walking-primer PCR (CDWP-PCR), is presented in this study. CDWP-PCR involves adopting a center degenerated walking primer (cdWP) in the secondary/tertiary round of amplification. This cdWP is generated by degenerating the seven central nucleotides of the normal walking primer (nWP) used in primary PCR to NNNNNNN (where N includes the bases A, T, C, and G). Clearly, a partially complementary structure is formed between the two primers. Accordingly, the primary CDWP-PCR non-target products defined by the nWP are diluted in secondary/tertiary CDWP-PCR, as these non-targets have difficulty in annealing with the cdWP; conversely, the primary target product can still be efficiently amplified. The working performance of the proposed CDWP-PCR is verified through cloning of the unknown flanks of three known genes. All the clear DNA bands in the tertiary CDWP-PCRs are confirmed to be correct, and the largest DNA band is 8.0 kb. Overall, CDWP-PCR can be considered as a reliable supplement to existing genome-walking methods.

## 1. Introduction

Genome walking is a kind of molecular tool which facilitates access to unknown flanking genomic sequences [1,2,3,4]. The availability of a short, known DNA sequence is the only prerequisite for genome walking [5,6,7,8]. Genome walking has been used in numerous areas associated with biology, such as investigating retroviruses, revealing transposable factors, and mining genetic regulatory elements [9,10,11,12,13]. Genome walking has thus greatly promoted the development of various biological fields. Although third-generation DNA sequencing technology has emerged in recent years, genome walking still holds a place in the field of biology due to its convenience and low cost [14,15,16,17].

A variety of genome-walking approaches are now available [10,18]. Among these approaches, those relying on genomic DNA libraries are now outdated, given their rather complicated and time-consuming nature. Relatively speaking, PCR-based genome-walking approaches are far more popular, which can be attributed to their ease of use, high speed, and low cost [15]. However, it is worth noting that the reported PCR-based approaches differ significantly in terms of methodological principles and processes, which are mainly related to whether the pretreatment of genomic DNA is performed [19,20].

An earlier PCR genome-walking approach requires pretreating genomic DNA, which includes endonuclease digestion of genomic DNA and subsequently cyclizing the treated DNA or ligation with a cassette/adaptor/linker [10,21]. Panhandle PCR [22,23,24], T-linker PCR [9,25], and inverse PCR [26,27] are considered to be such approaches. Later, PCR methods that do not require genomic DNA pretreatment—namely, random PCRs—were invented sequentially for genome walking [3,10]. These random PCR methods do not require a tedious genomic DNA pretreatment process and, thus, have gradually become the mainstream approach to genome walking. Thermal asymmetric interlaced PCR [3,28], uracil walking-primer PCR [20], and wristwatch PCR [29] are classical representatives of random PCR methods. However, these methods may be further improved in terms of the specificity or operability of the amplification process [30].

We herein propose the center degenerated walking-primer PCR (CDWP-PCR) method to enable efficiently and accurately executed genome walking. CDWP-PCR is characterized by the use of a center degenerated walking primer (cdWP) in the secondary/tertiary round of amplification. In particular, this cdWP is derived from the normal walking primer (nWP) used in primary CDWP-PCR, and is generated by degenerating the seven central nucleotides of the nWP into NNNNNNN (where N includes A, T, G, and C). Consequently, secondary/tertiary CDWP-PCR amplification of non-target DNA defined by the nWP is mitigated, as this DNA is prone to forming a hairpin via the nWP ends rather than binding to cdWP [31,32]; however, the target DNA can still be efficiently boosted through the proposed approach. The working performance of CDWP-PCR is verified through the cloning of unknown flanks of three known genes.

## 2. Materials and Methods

### 2.1. Genomic Templates

The genomic DNA of *Levilactobacillus brevis* was extracted using a Fastpure Bacteria DNA Isolation Mini Kit obtained from Vazyme Biotech Co., Ltd. (Nanjing, China). The rice genome used in this study was donated by Dr. Xiaojue Peng at Nanchang University, China.

### 2.2. Design of Primer

We designed three WP (walking primer) sets, aiming to execute three parallel CDWP-PCRs for each genome-walking experiment. Each WP set consisted of the nWP and its corresponding cdWP. The sequence of the nWP was arbitrary, while its corresponding cdWP had a fully degenerated central region (7 nt) and unchanged 5′ (about 10 nt) and 3′ (5 nt) regions (Table 1).

A set of three gene-specific primers (GSPs) (outmost GSP [oGSP], middle GSP [mGSP], and innermost GSP [iGSP]) was selected from the gene of hygromycin (*hyg*), glutamate tRNA ligase (*gluT*), or a hypothetical protein (*hyp*), respectively (Table 1).

### 2.3. Procedure of CDWP-PCR

An nWP was utilized with oGSP to perform primary CDWP-PCR, with genomic DNA used as a template; meanwhile, the corresponding cdWP was utilized with mGSP or iGSP to perform secondary and tertiary CDWP-PCR, respectively. In secondary or tertiary CDWP-PCR, the product of the former round of CDWP-PCR was used as a template.

The primary CDWP-PCR solution (50 μL) consisted of the following components: 0.4 μL of 10–100 ng/μL bacterial genome or 100–1000 ng/μL rice genome, 5 μL of 10 × LA Taq Buffer II (Mg^2+^ Plus), 4 μL of 2.5 mM each dNTP, 1 μL of 10 μM oGSP, 1 μL of 10 μM nWP, and 2.5 U of TaKaRa LA Taq HS polymerase. The PCR was completed via a two-step cycling program: step i—one 25 °C annealing cycle; step ii—forty 50 °C cycles (Table 2).

The secondary CDWP-PCR solution (50 μL) consisted of the following components: 0.4 μL of primary CDWP-PCR product, 5 μL of 10 × LA Taq Buffer II (Mg^2+^ Plus), 4 μL of 2.5 mM each dNTP, 1 μL of 10 μM mGSP, 1 μL of 10 μM cdWP, and 2.5 U of TaKaRa LA Taq HS polymerase. The PCR was completed via thirty 50 °C cycles (Table 2).

The tertiary CDWP-PCR solution (50 μL) consisted of the following components: 0.4 μL of secondary CDWP-PCR product, 5 μL of 10 × LA Taq Buffer II (Mg^2+^ Plus), 4 μL of 2.5 mM each dNTP, 1 μL of 10 μM iGSP, 1 μL of 10 μM cdWP, and 2.5 U of TaKaRa LA Taq HS polymerase. The PCR was completed via thirty 50 °C cycles (Table 2).

### 2.4. Effects of Degeneracy of cdWP

To determine the proper degeneracy of cdWP, four cdWP1s (GGCTATGAAAACAGAACTCGACCTC [also nWP1], GGCTATGAAAACAGANNNNNACCTC, GGCTATGAAAACANNNNNNNACCTC, and GGCTATGAAANNNNNNNNNNACCTC) were individually tested using *gadA* as the walking object. Please note that the four cdWP1s had 0, 5, 7, and 10 degenerate nucleotides, respectively. *gadA* was selected because it is an essential gene responsible for decarboxylating L-glutamate to gamma-aminobutyric acid and has been well studied [33]. The used oGSP, mGSP, and iGSP were AGCAGCCGTAACTTCTCCCACTAAG, TCCATACCCTCATCTCCATTTCCAT, and AACTATCACCCCACAACGTCATCTC, respectively. After primary CDWP-PCR had been completed with the oGSP and nWP1, the four cdWP1s were individually paired with mGSP or iGSP to perform secondary or tertiary CDWP-PCRs.

### 2.5. DNA Sequencing and Analysis

We performed 1% agarose gel electrophoresis to analyze the CDWP-PCR products. The secondary and tertiary amplicons of prominence were recovered with a DiaSpin DNA Gel Extraction kit obtained from Sangon Biotech Co., Ltd. (Shanghai, China). The recovered DNA was mailed to Sangon Biotech Co., Ltd., where it was directly sequenced. The obtained DNA sequence information was analyzed following the method previously described by Sun et al. [34].

## 3. Results

### 3.1. Overview of CDWP-PCR

**Figure 1:**
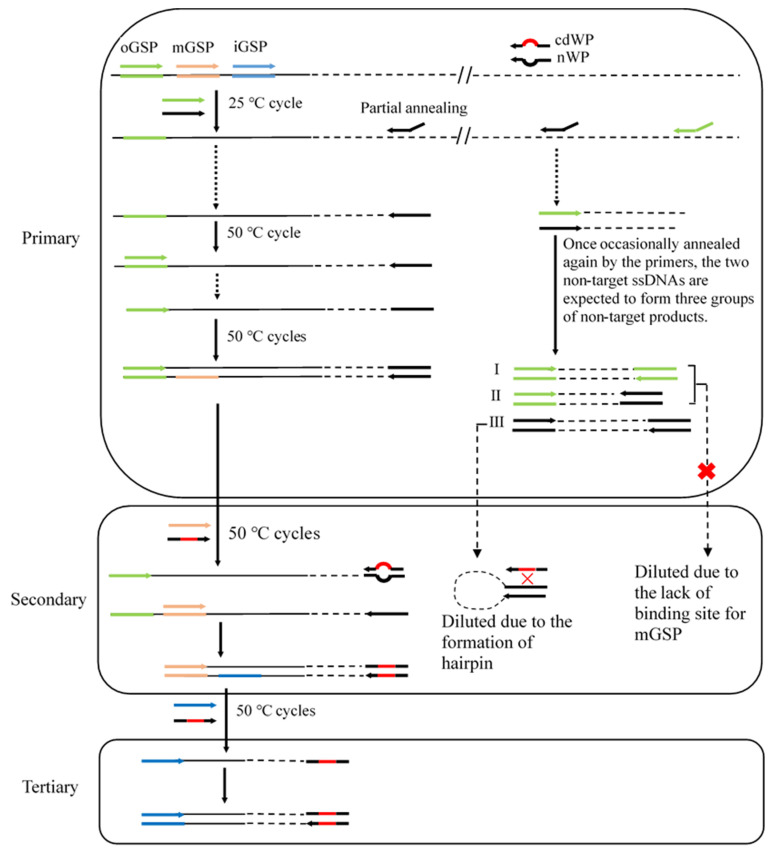
Schematic diagram of CDWP-PCR. CDWP-PCR: center degenerated walking-primer PCR; black thin solid line: known sequence; black thin dashed line: unknown sequence; arrows: primers; thick solid lines: primer complements; oGSP: outmost gene-specific primer; mGSP: middle gene-specific primer; iGSP: innermost gene-specific primer; nWP: normal walking primer; cdWP: center degenerated walking primer, with 7 central nucleotides being NNNNNNN (N including the bases A, T, C, and G) that are indicated by red line.

The CDWP-PCR set comprised three successive nested amplifications, namely primary, secondary, and tertiary amplifications (Figure 1 and Supplementary File S1).

In primary CDWP-PCR, the 25 °C cycle allowed nWP to partially hybridize with some location(s) in unknown flanking regions, while allowing nWP and oGSP to partially hybridize with many other locations on the genome. As a result, a target ssDNA (single-stranded DNA) and many non-target ssDNAs were synthesized, and they were then utilized as a template in secondary CDWP-PCR. The non-target ssDNAs were theoretically deprived of the opportunity for amplification as they lacked a perfect binding site for any primers. However, three groups of non-target products—namely, group I defined by oGSP, group II defined by oGSP and nWP, and group III defined by nWP—were still expected to be produced once the non-target ssDNAs were annealed again by the primers.

In secondary CDWP-PCR, the primary CDWP-PCR product was utilized as a template. The target DNA could be efficiently amplified by mGSP and cdWP in this PCR step. Notably, group I and II non-target DNA could not be further amplified due to the lack of a true binding site for mGSP; group III non-target DNA was also difficult to amplify, as it was prone to forming a hairpin via the inverted nWP termini rather than hybridizing with the partially complementary cdWP.

Tertiary CDWP-PCR was performed identically to secondary CDWP-PCR, except that the template used was the secondary CDWP-PCR product and the GSP used was iGSP.

### 3.2. Effects of Degeneracy of cdWP

**Figure 2:**
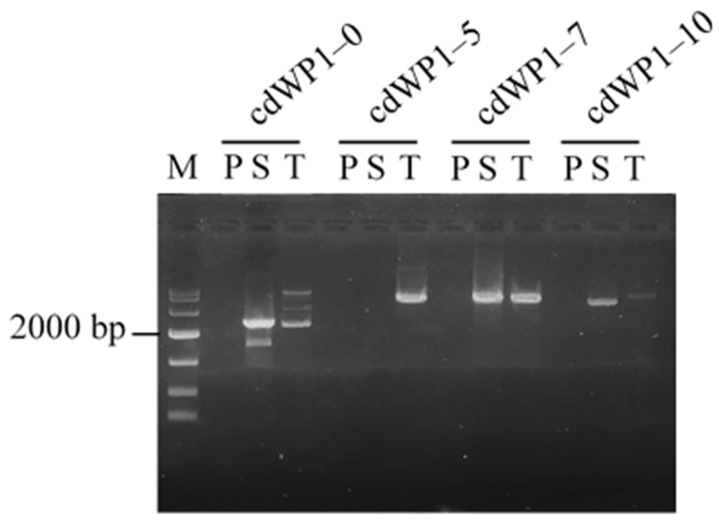
Effects of degeneracy of cdWP on CDWP-PCR. cdWP1–0, cdWP1–5, cdWP1–7, and cdWP1–10 represent the four genome-walking processes performed using cdWP1s with 0, 5, 7, and 10 degenerate nucleotides, respectively. The walked gene was *gadA*. CDWP-PCR: center degenerated walking-primer PCR; cdWP: center degenerated walking primer; P: primary CDWP-PCR; S: secondary CDWP-PCR; T: tertiary CDWP-PCR; M: DNA 10,000 marker (10,000, 7000, 4000, 2000, 1000, 500, and 250 bp).

The performances of the four cdWP1s are summarized in Figure 2. The secondary/tertiary CDWP-PCR results indicated that degenerating cdWP1 in 5–7 nucleotides was appropriate, as this resulted in satisfactory secondary/tertiary PCR amplification. Therefore, all cdWPs were degenerated in seven nucleotides for the following experiments. It should be pointed out that there was no PCR product after secondary PCR with cdWP1-5. We attribute this absence of product to the randomness of PCR.

### 3.3. Validation of CDWP-PCR

**Figure 3:**
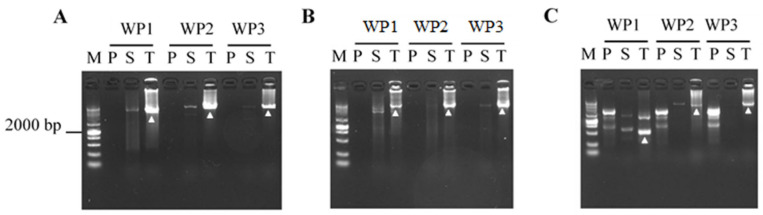
CDWP-PCRs of the genes *hyp* (**A**) and *gluT* (**B**) in *Levilactobacillus brevis* and *hyg* (**C**) in rice. WP1, WP2, and WP3 represent the three parallel sets of CDWP-PCRs in a walking experiment. CDWP-PCR: center degenerated walking-primer PCR; WP: walking primer; P: primary CDWP-PCR; S: secondary CDWP-PCR; T: tertiary CDWP-PCR; M: DNA 10,000 marker (10,000, 7000, 4000, 2000, 1000, 500, and 250 bp); *hyp*: hypothetical protein gene; *glut*: glutamate tRNA ligase gene; *hyg*: hygromycin gene.

To verify the working performance of CDWP-PCR, we used this technique to explore unknown segments flanking the three genes *gluT*, *hyp*, and *hyg*. The CDWP-PCR products were electrophoresed on a 1.0% agarose gel. As presented in Figure 3, all nine tertiary CDWP-PCRs released clear DNA bands. The sequencing data confirmed that these DNA bands were correct (Supplementary Figure S1). Interestingly, all tertiary CDWP-PCRs showed only one major PCR end-product. In addition, the walk distance of CDWP-PCR reached up to 8.0 kb.

## 4. Discussion

Over time, scientific communities have increasingly favored random PCR genome-walking approaches [35,36]. In such an approach, however, three groups of non-target DNA are always produced in primary PCR, which are generated by oGSP alone (group I), oGSP plus WP (group II), and WP alone (group III), respectively. Group I and II non-target DNA can be easily excluded through the inner nested GSP in the following PCR. However, how to exclude group III non-target DNA is a recognized challenge in the context of random PCR. Therefore, a truly practical random PCR should be able to efficiently enrich target DNA while efficiently reducing this non-target DNA [37,38].

Most random PCR methods rely on a set of three non-degenerate WPs with partial overlap. In secondary or tertiary PCR, the low-stringency cycle(s) allows WP to bind to the previous WP site on group III non-target DNA, thus newly synthesizing an ssDNA. Once annealed again by the WP, this new ssDNA becomes a perfect template of the WP, resulting in a non-target background [39]. Uracil walking-primer PCR relies on WP with an uracil base near the 3′ end. Although this PCR approach can fundamentally overcome group III non-target DNA product, it requires treatment of the primary PCR product with uracil DNA glycosylase [20,40,41]. In the proposed CDWP-PCR method, the special design of cdWP not only ensures the specificity of amplification, but also does not require any enzyme treatment process. In secondary/tertiary CDWP-PCR, the degeneracy of the cdWP ensures that the amplification efficiency of target DNA is consistently higher than that of non-target DNA. It can be concluded that the amplification specificity of CDWP-PCR is at least no lower than that of existing random PCR methods.

CDWP-PCR can effectively alleviate the multi-band phenomenon that commonly occurs in other random PCRs [19,20,42]. In this study, all the tertiary CDWP-PCRs produced one clear DNA band, which is likely related to the following two facts: first, due to the absence of low-stringency cycle(s), internal annealing of cdWP to target DNA [29] is evaded in secondary/tertiary CDWP-PCR; second, the degeneracy of cdWP discourages this primer from annealing with minority target DNAs [19].

The walking ability of CDWP-PCR is also quite satisfactory. As presented in Figure 3, in each walking experiment, a target DNA over 8.0 kb was obtained. This walking distance is much longer than that of other PCR methods (generally around 3.0 kb) [5,22,23,24]. This difference in walking ability can—at least partly—be attributed to whether low-stringency cycles are performed in secondary/tertiary amplification. Most other PCRs require at least one such cycle in secondary/tertiary amplification. This promotes the partial annealing of WP within target DNA, resulting in shorter target DNA [37,38]. In general, PCR prioritizes amplifying shorter DNA. Consequently, the full-length amplification of target DNA is inhibited [29]. In contrast, secondary/tertiary CDWP-PCR does not include such a low annealing temperature cycle and, thus, the internal annealing of cdWP to target product is thus efficiently evaded. Therefore, it can be expected that the full-length amplification of target DNA is highly likely to be achieved.

## 5. Conclusions

In this study, we proposed a CDWP-PCR-based genome-walking method. CDWP-PCR mainly relies on a specially designed cdWP. This primer can continuously inhibit the amplification of group III non-target DNA, thereby improving the specificity and efficiency of CDWP-PCR-based amplification. The working performance of CDWP-PCR was confirmed through the extension of unknown flanks of several known genes. The proposed CDWP-PCR can be considered as a reliable supplement to existing genome-walking PCRs.

## Figures and Tables

**Table 1:** Primers utilized in this study.

Primer Set ^a^	Primary CDWP-PCR	Secondary CDWP-PCR	Tertiary CDWP-PCR
hyp	CCAAGGACTTAGGCTTTGACATTGA	GGATGCTGCCTTCGGTGGGTTATTT	TGGTCACAAGTACGGCATGGTTTAC
gluT	TCCTTCGTTCTTGATTCCATACCCT	CCATTTCCATAGGTTGCTCCAAGG	GGATACTGGCTAAAATGAATTAACTCGGATAA
hyg	TTTCAGCTTCGATGTAGGAGGGCGT	GTAAATAGCTGCGCCGATGGTTTCT	TTGACATTGGGGAGTTTAGCGAGAG
WP1	GGCTATGAAAACAGAACTCGACCTC	GGCTATGAAAACANNNNNNNACCTC	GGCTATGAAAACANNNNNNNACCTC
WP2	CCTGACCGCCTTCTACACCT	CCTGACCGNNNNNNNCACCT	CCTGACCGNNNNNNNCACCT
WP3	ATGCTGCTCGTGGATGACTCT	ATGCTGCTCNNNNNNNACTCT	ATGCTGCTCNNNNNNNACTCT

^a^ The three gene-specific primer (GSP) sets were hyp, gluT, and hyg. A GSP set comprised three nested GSPs that were sequentially used in primary, secondary, and tertiary CDWP-PCRs. The three walking primer (WP) sets were WP1, WP2, and WP3. A WP set comprised the nWP (normal walking primer) used in primary CDWP-PCR, and the corresponding cdWP (center degenerated walking primer) used in secondary/tertiary CDWP-PCR. A GSP was paired with the three WPs in the same column to perform three parallel CDWP-PCRs.

**Table 2:** Thermal cycling program of CDWP-PCR.

Round of PCR	Thermal Parameters	Cycle Number
Primary	94 °C, 2 min	1
	94 °C, 30 s; 25 °C, 30 s; 72 °C, 2 min	1
	94 °C, 30 s; 50 °C, 30 s; 72 °C, 2 min	40
	72 °C, 5 min	1
Secondary	94 °C, 2 min	1
	94 °C, 30 s; 50 °C, 30 s; 72 °C, 2 min	30
	72 °C, 5 min	1
Tertiary	94 °C, 2 min	1
	94 °C, 30 s; 50 °C, 30 s; 72 °C, 2 min	30
	72 °C, 5 min	1

## Data Availability

Gene information from *L. brevis* CD0817 and rice can be downloaded from https://www.ncbi.nlm.nih.gov/genbank/ (accessed on 11 October 2023), AYM03982.1, and https://www.ncbi.nlm.nih.gov/genbank/, KF206149 (accessed on 11 October 2023).

## Supplementary Materials

The following supporting information can be downloaded at: https://www.mdpi.com/article/10.3390/cimb47080602/s1.
