# Supplementary material for: Center Degenerated Walking-Primer PCR: A Novel and Universal Genome-Walking Method
Source: Curr Issues Mol Biol. 2025 Aug 1;47(8):602. doi: 10.3390/cimb47080602 (PMC12384727; doi:10.3390/cimb47080602)
Supplement: Supplementary file 1 [file cimb-47-00602-s001.zip › Supplementary Figure S1.pdf]

hyp

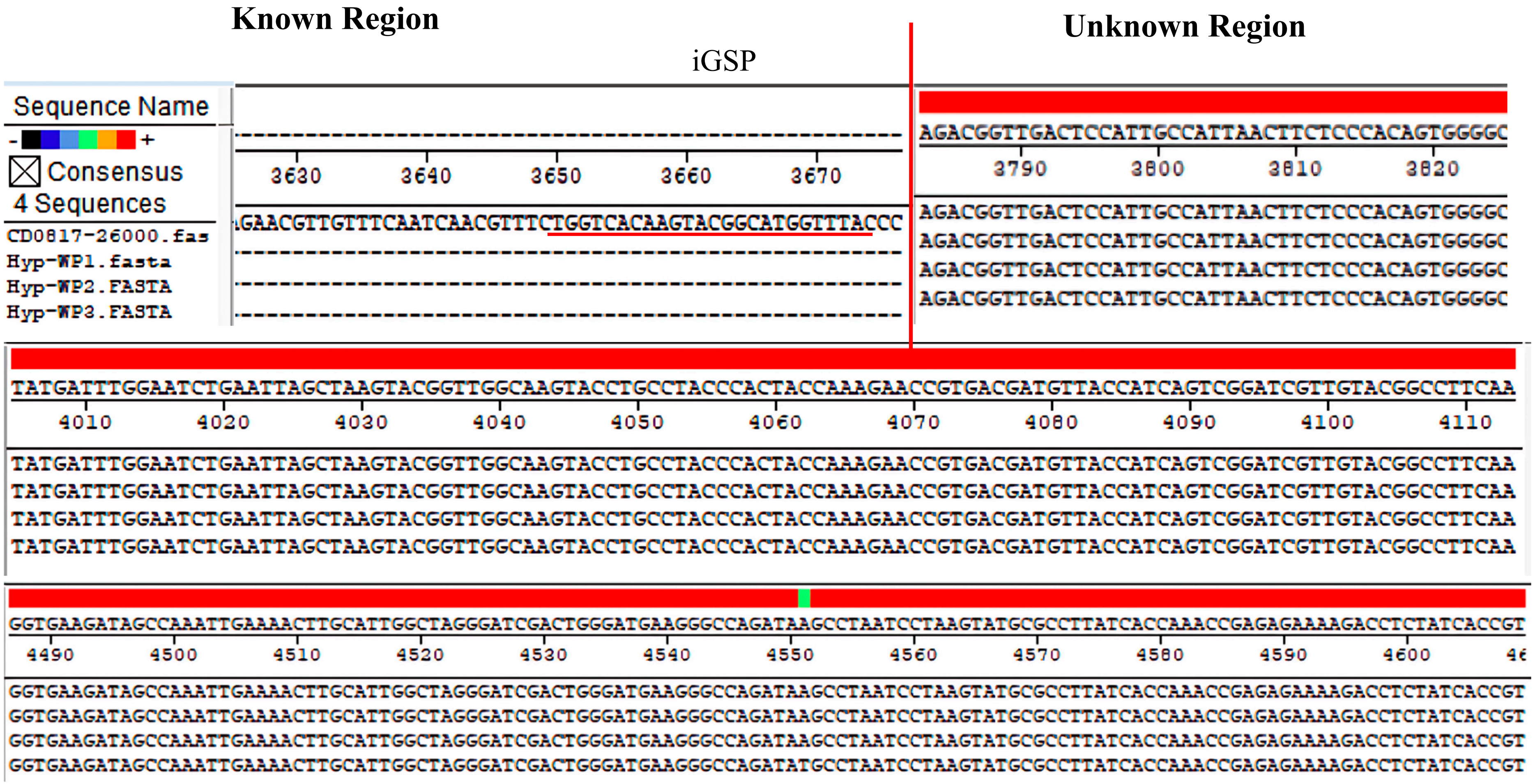
Known Region
iGSP
Unknown Region
Sequence Name
- +
Consensus
4 Sequences
CD0817-26000.fas
Hyp-WP1.fasta
Hyp-WP2.FASTA
Hyp-WP3.FASTA
3630 3640 3650 3660 3670
GAACGTTGTTTCAATCAACGTTTCTGGTCACAAGTACGGCATGGTTTACCC
AGACGGTTGACTCCATTGCCATTAACTTCTCCCACAGTGGGGC
3790 3800 3810 3820
AGACGGTTGACTCCATTGCCATTAACTTCTCCCACAGTGGGGC
AGACGGTTGACTCCATTGCCATTAACTTCTCCCACAGTGGGGC
AGACGGTTGACTCCATTGCCATTAACTTCTCCCACAGTGGGGC
AGACGGTTGACTCCATTGCCATTAACTTCTCCCACAGTGGGGC
TATGATTTGGAATCTGAATTAGCTAAGTACGGTTGGCAAGTACCTGCCTACCCACTACCAAAGAACCGTGACGATGTTACCATCAGTCGGATCGTTGTACGGCCTTCAA
4010 4020 4030 4040 4050 4060 4070 4080 4090 4100 4110
TATGATTTGGAATCTGAATTAGCTAAGTACGGTTGGCAAGTACCTGCCTACCCACTACCAAAGAACCGTGACGATGTTACCATCAGTCGGATCGTTGTACGGCCTTCAA
TATGATTTGGAATCTGAATTAGCTAAGTACGGTTGGCAAGTACCTGCCTACCCACTACCAAAGAACCGTGACGATGTTACCATCAGTCGGATCGTTGTACGGCCTTCAA
TATGATTTGGAATCTGAATTAGCTAAGTACGGTTGGCAAGTACCTGCCTACCCACTACCAAAGAACCGTGACGATGTTACCATCAGTCGGATCGTTGTACGGCCTTCAA
TATGATTTGGAATCTGAATTAGCTAAGTACGGTTGGCAAGTACCTGCCTACCCACTACCAAAGAACCGTGACGATGTTACCATCAGTCGGATCGTTGTACGGCCTTCAA
GGTGAAGATAGCCAAATTGAAAACTTGCATTGGCTAGGGATCGACTGGGATGAAGGGCCAGATAAGCCTAATCCTAAGTATGCGCCTTATCACCAAACCGAGAGAAAAGACCTCTATCACCGT
4490 4500 4510 4520 4530 4540 4550 4560 4570 4580 4590 4600
GGTGAAGATAGCCAAATTGAAAACTTGCATTGGCTAGGGATCGACTGGGATGAAGGGCCAGATAAGCCTAATCCTAAGTATGCGCCTTATCACCAAACCGAGAGAAAAGACCTCTATCACCGT
GGTGAAGATAGCCAAATTGAAAACTTGCATTGGCTAGGGATCGACTGGGATGAAGGGCCAGATAAGCCTAATCCTAAGTATGCGCCTTATCACCAAACCGAGAGAAAAGACCTCTATCACCGT
GGTGAAGATAGCCAAATTGAAAACTTGCATTGGCTAGGGATCGACTGGGATGAAGGGCCAGATAAGCCTAATCCTAAGTATGCGCCTTATCACCAAACCGAGAGAAAAGACCTCTATCACCGT
GGTGAAGATAGCCAAATTGAAAACTTGCATTGGCTAGGGATCGACTGGGATGAAGGGCCAGATATGCCTAATCCTAAGTATGCGCCTTATCACCAAACCGAGAGAAAAGACCTCTATCACCGT

gluT

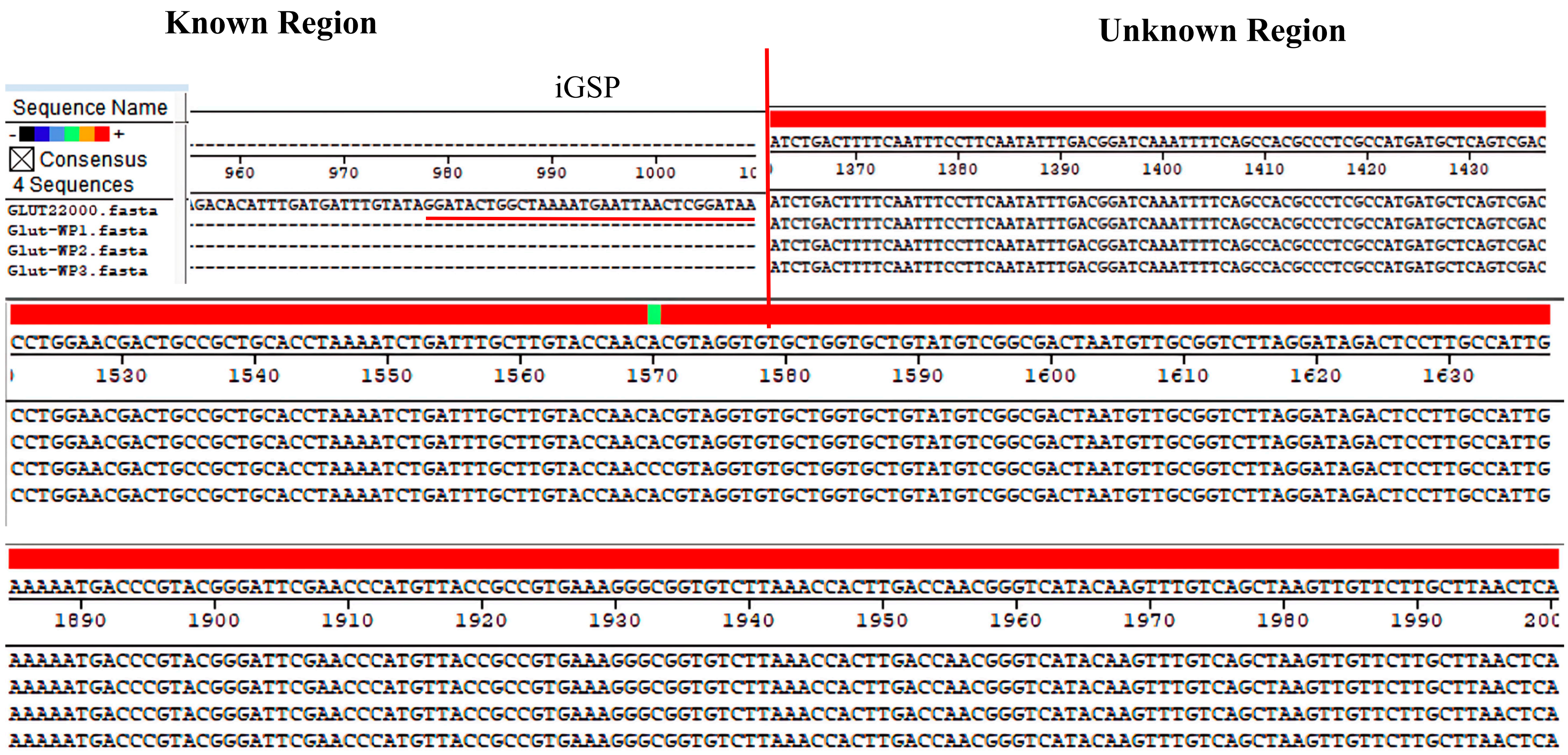
Known Region
Unknown Region
iGSP
Sequence Name
Consensus
4 Sequences
GLUT22000.fasta
Glut-WP1.fasta
Glut-WP2.fasta
Glut-WP3.fasta
GACACATTTGATGATTTGTATAGGATACTGGCTAAAATGAATTAACTCGGATAA
ATCTGACTTTTCAATTTCCTTCAATATTTGACGGATCAAATTTTCAGCCACGCCCTCGCCATGATGCTCAGTCGAC
CCTGGAACGACTGCCGCTGCACCTAAAATCTGATTTGCTTGTACCAACACGTAGGTGTGCTGGTGCTGTATGTCGGCGACTAATGTTGCGGTCTTAGGATAGACTCCTTGCCATTG
AAAAATGACCCGTACGGGATTCGAACCCATGTTACCGCCGTGAAAGGGCGGTGTCTTAAACCACTTGACCAACGGGTCATACAAGTTTGTCAGCTAAGTTGTTCTTGCTTAACTCA

Hyg

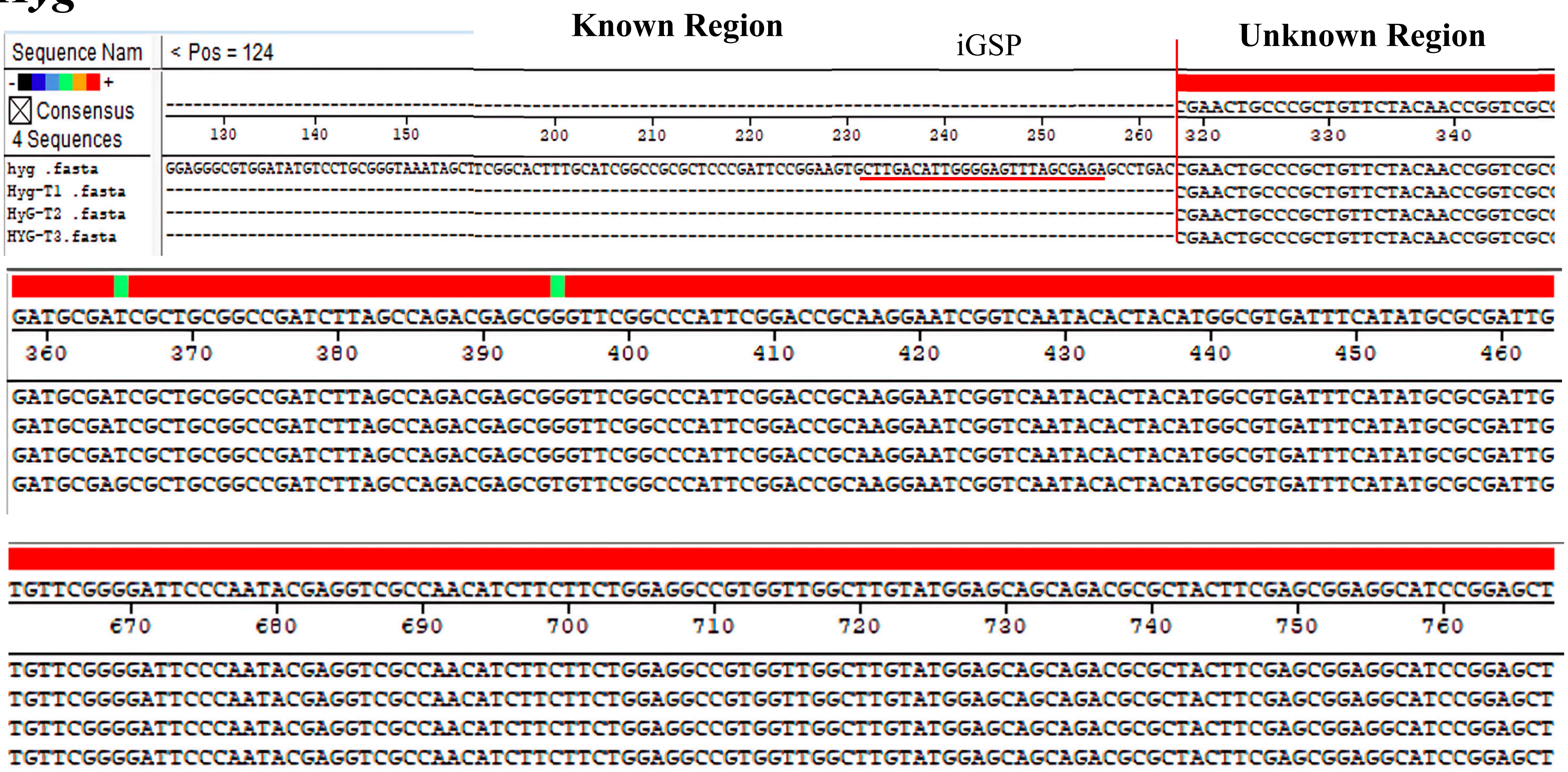

Supplementary Figure S1. Analysis of sequencing data of the walking results.
