# Supplementary material for: Center Degenerated Walking-Primer PCR: A Novel and Universal Genome-Walking Method
Source: Curr Issues Mol Biol. 2025 Aug 1;47(8):602. doi: 10.3390/cimb47080602 (PMC12384727; doi:10.3390/cimb47080602)
Supplement: Supplementary file 1 [file cimb-47-00602-s001.zip › Supplementary File S1. Animation Demonstration.pptx]

## Slide 1
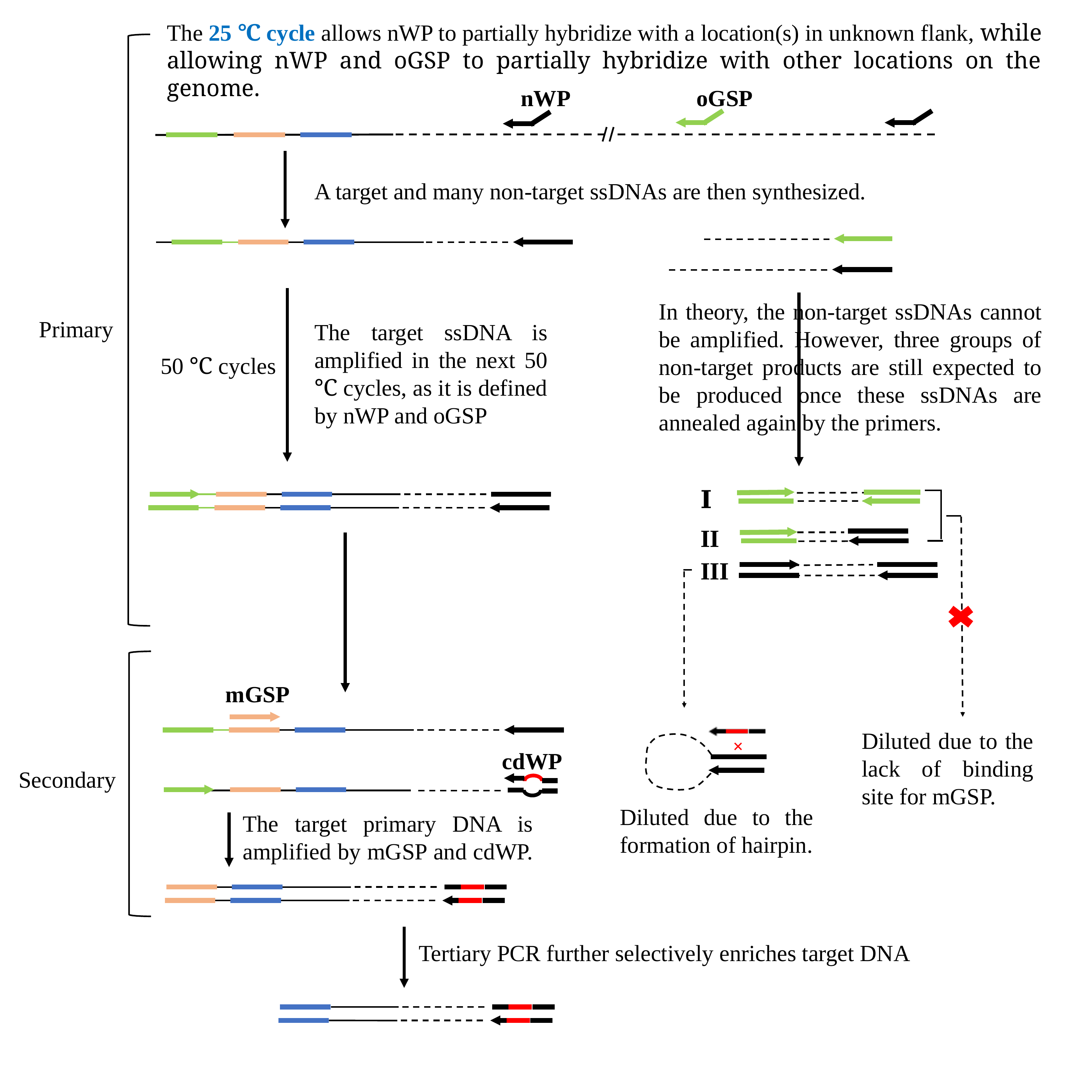

The 25 ℃ cycle allows nWP to partially hybridize with a location(s) in unknown flank, while allowing nWP and oGSP to partially hybridize with other locations on the genome.
Primary
nWP
oGSP
A target and many non-target ssDNAs are then synthesized.
50 ℃ cycles
Ⅰ
II
III
In theory, the non-target ssDNAs cannot be amplified. However, three groups of non-target products are still expected to be produced once these ssDNAs are annealed again by the primers.
The target ssDNA is amplified in the next 50 ℃ cycles, as it is defined by nWP and oGSP
Diluted due to the lack of binding site for mGSP.
×
Diluted due to the formation of hairpin.
mGSP
cdWP
The target primary DNA is amplified by mGSP and cdWP.
Secondary
Tertiary PCR further selectively enriches target DNA
